# The rare mutation in the endosome-associated recycling protein gene *VPS50* is associated with human neural tube defects

**DOI:** 10.1186/s13039-019-0421-9

**Published:** 2019-02-20

**Authors:** Zhiwen Shi, Shuxia Chen, Xiao Han, Rui Peng, Jin Luo, Luming Yang, Yufang Zheng, Hongyan Wang

**Affiliations:** 1 Obstetrics and Gynecology Hospital, State Key Laboratory of Genetic Engineering at School of Life Sciences, Institute of Reproduction and Development, Fudan University, Shanghai, 200011 China; 2 Key Laboratory of Reproduction Regulation of NPFPC, Collaborative Innovation Center of Genetics and Development, Fudan University, Shanghai, 200032 China; 3 Institute of Developmental Biology & Molecular Medicine, Fudan University, Shanghai, 200433 China; 4 Children’s Hospital and Institutes of Biomedical Sciences of Fudan University, 399 Wanyuan Road, Shanghai, 201102 China

**Keywords:** *VPS50*, EARP, WISH, Zebrafish, NTDs

## Abstract

**Background:**

Tight control of endosome trafficking is essential for the generation of a normally patterned embryo. Recent studies have found that VPS50 is a key ingredient in EARP which is required for recycling of internalized TfRs to the cell surface and dense-core vesicle maturation. However, the role of *VPS50* in embryogenesis and human physiology are poorly understood.

**Results:**

We identified a rare missense heterozygous *VPS50* mutation (p. Gly169Val) in NTDs by high-throughput sequencing. In vitro functional analysis demonstrated that the p. Gly169Val was a loss-of-function mutation, delaying transferrin recycling and altering its interaction with VPS53. Using WISH during zebrafish embryogenesis, we demonstrated that *vps50* gene was expressed throughout the early embryo, especially in the head. Abnormal body axis phenotypes were observed in those *vps50* knock-down zebrafishes. Further rescue study in zebrafish suggested that the mutation displayed loss-of-function effects comparing with wild-type *VPS50*.

**Conclusions:**

These findings thus demonstrated that the functional mutations in *VPS50* might contribute to neurodevelopmental disorder and highlighted the critical importance of *VPS50* function in cellular and organismal physiology.

**Electronic supplementary material:**

The online version of this article (10.1186/s13039-019-0421-9) contains supplementary material, which is available to authorized users.

## Background

Endosomes, mediating transport of intracellular and extracellular cargos, are important functional elements in the organization of intracellular membrane dynamics [1]. In addition to the house-keeping functions common to all eukaryotic cells, endosomal transport appears to be of particular importance in polarized cell types such as the extension of axons and dendrites in neuronal cells [2]. For simplicity’s sake, endosomes’ components are frequently dismantled into discrete units such as early (sorting) endosomes, late endosomes (multivesicular bodies), recycling endosomes and lysosomes [3–5]. Once in endosomes, the fates of internalized proteins diverge. Some proteins are transported to lysosomes, other internalized proteins are delivered to the trans-Golgi network or back to the plasma membrane by recycling endosomes.

In general, recycling endosomes are found associated with unique sets of proteins [6]. Recent studies show that EARP is involved in recycling cargos from endosomes back to plasma membrane. It shares three identical proteins with GARP: Vps51, Vps52 and Vps53 [7], but uses VPS50 instead of VPS54 as the fourth subunit [8, 9]. This change determines differential localization of EARP to recycling endosomes and GARP to the Golgi complex. VPS50 is mainly associated with the cytosolic face of endosomes marked by the small GTPase Rab4, and promotes the recycling of TfRs from endosomes to the plasma membrane. The functions of EARP in TfRs transport are essential for the viability of mammalian organisms, as demonstrated by the embryonic lethality of mice with homozygous null mutations in this pathway, showing abnormalities in the nervous system such as kinked neural tubes [10–12]. Recent genome-wide linkage analysis also identified mutations in *VPS53* and *VPS51* genes encoding different subunits of EARP in patients with complex neurological diseases [13, 14]. These results raise the possibility that rare mutations in *VPS50* may be associated with human NTDs.

## Material and methods

### Study subjects and capture DNA sequencing

100 patients with NTDs and 171 healthy controls were all ethnically Han Chinese (Table 1). All the cases were collected in Shanxi Province. The unrelated healthy controls (*n* = 171,17.9 ± 1.3 years) were collected from new recruits in Shandong and Henan province of China. Each sample was collected with the approval of the local ethics committee and institutional review board of Fudan University. Written consent was obtained from the patients’ parents. Whole genome sequencing was conducted using Illumina HiSeq X ten at WuXi AppTec. Genotyping was performed according to the Infinium HD protocol from Illumina. All controls were confirmed by Sanger sequencing as previously described [15]. Partial alignment of Vps50 amino acid sequences between human and other vertebrates was aligned using the Clustalx program. The effect of missense variants were predicted according to SIFT [16] (Sorting Intolerant from Tolerant; http://sift.jcvi.org/) and PolyPhen-2 [17] (Polymorphism Phenotyping version 2.1.0; http://genetics.bwh.harvard.edu/pph/) programs.

**Table 1:** Demographic Characteristics in NTD Cohor

Variable	Case (%)	Control (%)
Gender		
Male	60 (60%)	0
Female	37 (37%)	171(100%)
Unknow	3 (3%)	0
Case type		
AE	95(95%)	0
CRS	66(95%)	0
OEC	33(20%)	0
MM	1(1%)	0
Age:years/weeks (mean ± SD)		
Case	20.7 ± 4.7(weeks)	
Control	24.40 ± 2.87y	

*AE* anencephaly, *CRS* craniorachischisis, *OEC* occipital encephalocele, *MM* myelomeningocele

### Plasmid constructs and in vitro transcription of amplified cDNA

Human *VPS50* cDNA (NM_017667) was cloned into pEnter-Flag/His vector with puromycin screening marker. The plasmid constructs were verified by Sanger sequencing. The *VPS50* vectors were linearized with XhoI restriction enzyme (NEB, USA) and transcribed with the T7 mMESSAGE mMACHINE kit (Ambion, USA). The reaction was carried out at 37 °C for 1 h, followed by the addition of DNase I and incubation for 15 min. Ammonium acetate was added, and RNA was isolated by phenol/chloroform extraction and isopropanol precipitation. After centrifugation, the RNA pellet was resuspended in RNase-free water, and purity was determined by UV spectrophotometry and electrophoresis.

### Generation of *VPS50* knockout HeLa cell lines

For *VPS50* KO Hela cell lines, the gRNA was designed and cloned in an U6 targeting vector [18] and the single clones were established by dilution cloning. Knockout efficiencies were confirmed by Western Blotting and Immunofluorescence. gRNA sequence used was:

VPS50 exon1 5’-CAAATCTCTCATGACCCGAC-3’ VPS50 KO.

### Western blot

Cells were lysed in RIPA buffer (Beyotime, China). The lysates were denatured at 100 °C for 5 min and then cooled down on ice. Then lysates were loaded on sodium dodecyl sulfatepolyacrylamide gel (SDS-PAGE) (10%) and electrotransferred onto polyvinylidene difluoride (PVDF) membrane. After blocking with 5% nonfat milk in TBST (Tris-buffered saline, 0.1% Tween 20) for 2 h at room temperature, PVDF membranes were blotted with primary antibody at 4 °C for 12 h, then incubated with HRP-labeled secondary antibody (CST, USA) at room temperature for 2 h. The bands were visualized using Tanon 5200 (Tanon, China). Primary antibodies are as follows: mouse monoclonal antibody to VPS50 (Abnova, China). Mouse monoclonal antibody to beta-actin (CST, USA).

### Co-immunoprecipitation

HEK293T cells were transfected with 4μg wild-type or the VPS50 mutation and 4μg VPS53-HA vector per well using Lipofectamine2000 reagent (Life Technologies, USA) by manufacturer’s instructions with a 0.5:1 ratio of reagent to DNA. Cells were allowed to incubate with transfection media for 48 h then were washed with PBS and harvested in cold RIPA lysis buffer (Beyotime, China) containing protease inhibitors (Roche, Germany). Cells were lysed by rocking at 4 °C for 20 min and then Immunoprecipitation with anti-HA mouse polyclonal agarose beads (Abmart, China).

### Immunofluorescence and transferrin chase

Immunofluorescence was performed as described previously [19] on a Zeiss LSM700 microscope (Carl Zeiss, Germany).Transferrin chase was carried out using a modification of a previously described protocol [8].

### Whole mount in situ hybridization

A 626 bp cDNA fragment of *vps50* was sub cloned into pGEM-T-easy vector (Promega, USA), which was conservative across species, with the primers as the following: 5’-GGCAGCCAAAGCCATAGA-3′ (forward); 5’-TGCAGACCACGCAAGACA-3′ (reverse). Spe I and Nco I restriction enzyme (NEB, USA) were chosen to linearize the probe plasmid respectively. The Digoxigenin-labeled sense and antisense probes were synthesized by Sp6 and T7 mMESSAGE mMACHINE kit (Ambion, USA). The wild type (WT) AB zebrafish embryos were collected and fixed with 4% paraformaldehyde (PFA) in phosphate-buffered saline (PBS) in the 4 °C for overnight. Then the embryos were dehydrated with gradient Methanol and PBST (Tween-20, 1‰) mixture and stored in 100% Methanol in − 20 °C. Whole mount in situ hybridization was subsequently followed the protocol designed by Rodney M. Dale’s study [20].

### Zebrafish embryo microinjections

The WT AB zebrafish were maintained and bred under standard conditions and embryo microinjection was performed following the standard protocol [21]. Morpholino oligos (MOs, Gene Tools) acted as a means to inhibit gene function in embryos [22]. We injected *vps50* MOs, directed against the ATG of the vps50 gene, with a different concentration gradient, respectively, into zebrafish embryos during the one to two cell stages. To validate MOs knockdown efficiencies and verify the conservation of *Vps50* function, we injected human mRNA of *VPS50* with a different concentration gradient in pairwise combinations with MOs, respectively, into zebrafish embryos during the one to two cell stages. Post injection (48 h), embryos were observed by microscopy, phenotypes were scored, and images were taken using a QImaging microscope system. Zebrafish *vps50* MOs and standard control MOs were purchased from Gene Tools and the sequences were:

*vps50* MOs: 5′ –TTCTGCATCGAACAGTCGAGGACAC- 3′,

Control MOs: 5′ –CCTCTTACCTCAGTTACAATTTATA- 3’.

## Results

### Identification of *VPS50* mutations in NTDs

**Fig. 1:**
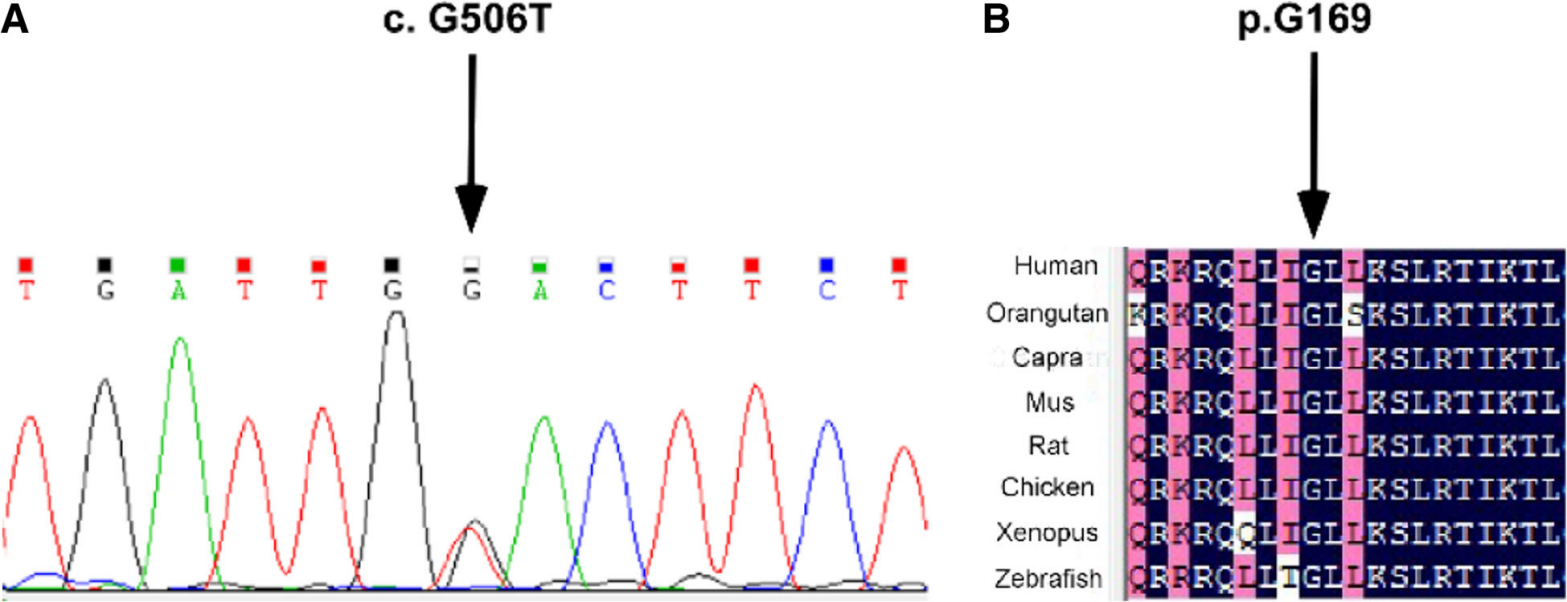
**a** Electropherograms showed the putative heterozygous mutation**.** The change in transcript sequences was depicted. **b** Alignment of VPS50 ortholog protein sequences using the ClustalW method**.** Multiple sequence alignment of the VPS50 indicated that p. Gly169 was highly evolutionarily conserved in vertebrates. Conserved residues were shaded by GeneDoc. The following sequences were used: human, NP_060137.2; *Danio rerio* (zebrafish), XP_017207797.1; Xenopus tropicalis (frog), NP_001107716.1; *Mus musculus* (mouse), NP_077222.4; *Capra hircus* (caprine), XP_005678979.1; Orangutan, XP_002818304.1; Rat, NP_001166982.1; Chicken, NP_001012872

Sequencing of *VPS50* revealed a heterozygous missense mutation c. G506 T (p. Gly169Val) in NTDs. The p. Gly169Val mutation was absent in our controls and the allele frequency of p. Gly169 allele was extremely low in the ExAc database (Table 2). By alignment of VPS50 ortholog protein sequences, we found that the p. Gly169 residue was highly conserved between species (Fig. 1b). Sanger sequencing is the gold standard for confirmation of minor variants detected by NGS (Fig. 1a).  A more detailed sequencing results of *VPS50* can be found in Additional file 1.

**Table 2:** Genotypes and Clinical Phenotypes Carrying the Mutations of *VPS50*

Nucleotide change^a^	Amino acid change^b^	Case no.	Control no.	SIFT^c^	PolyPhen2^d^	Sex	Age (weeks)	NTD type	MAF in cohort	MAF in CHB
c.G506 T	p. Gly169Val	1	0	0.04	1	F	12	anencephalus	0.001845	not reported
c.A1104T	p. Glu368Asp	1	1	0.25	0.003	F	24	anencephalus	0.00369	0.0049

^a^For nucleotide numbering, + 1 corresponds to the A of ATG of mRNA sequence 2. NM_017667.3

^b^Reference protein sequence NP_060137.2

^c^The threshold for intolerance is 0.05 and ranges from 0 to 1. The amino-acid substitution was predicted to be damaging if the score was <=0.05 and tolerated if the score was > 0.05

^d^The score is from 0 to 1; the amino-acid substitution was appraised qualitatively as benign or damaging based on pairs of false positive rate thresholds and optimized separately for each model

### The p. Gly169Val mutation delayed transferrin recycling

**Fig. 2:**
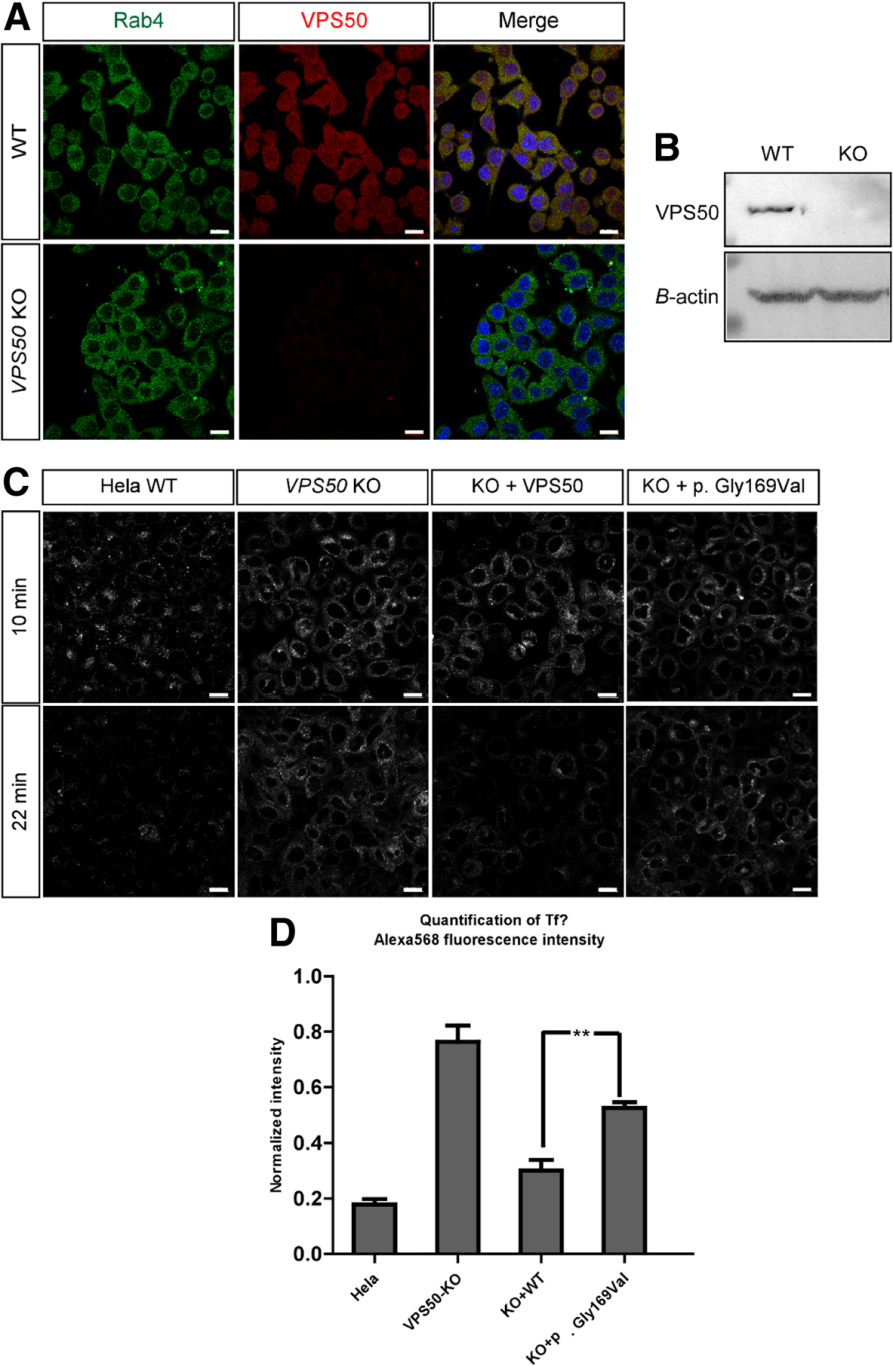
**a** and **b** Establish VPS50 knockout cell line**.** The immunofluorescence and western blotting showed that the VPS50 was totally knocked out in Hela cells. **c** and **d** p. Gly169Val mutation delayed Transferrin recycling**.** Transferrin was lost more rapidly in Hela cells than in *VPS50-KO* cells and the p. Gly169Val mutation followed the same pattern of delaying as *VPS50-KO* cells. 4μg wild-type or different VPS50 mutations were transfected into *VPS50-KO* cells. After 24 h, puromycin was added into the DMEM for 24 h to filter untransfected cells. Transferrin chase was carried out using a modification of a previously described protocol. Inset bar, 20um (∗∗*P* < 0.01, two-tailed t-test)

To investigate the in vitro activity of the p. Gly169Val mutation, we generated *VPS50* knockout Hela Cell Lines. Knockout efficiency was examined by Western Blot and Immunofluorescence (Fig. 2a and b). Then *VPS50*-KO Hela cells were transfected using Lipofectamine 3000 with various *VPS50* plasmid constructs. After 24 h, puromycin was added into the DMEM for 24 h to filter untransfected cells (Additional file 2: Figure S1). Then cells were subjected to transferrin chase. As previously described [8], the intracellular Alexa Fluor 568 Conjugated transferrin was lost more rapidly in Hela cells than in *VPS50-KO* cells and the p. Gly169Val mutation followed the same pattern of delaying transferrin recycling as the *VPS50*-KO cells.(Fig. 2c and d). These experiments thus demonstrated that the p. Gly169Val mutation partially disrupted the function of VPS50, delaying transferrin recycling.

### The p. Gly169Val mutation disrupted the interaction of VPS50 with VPS53

**Fig. 3:**
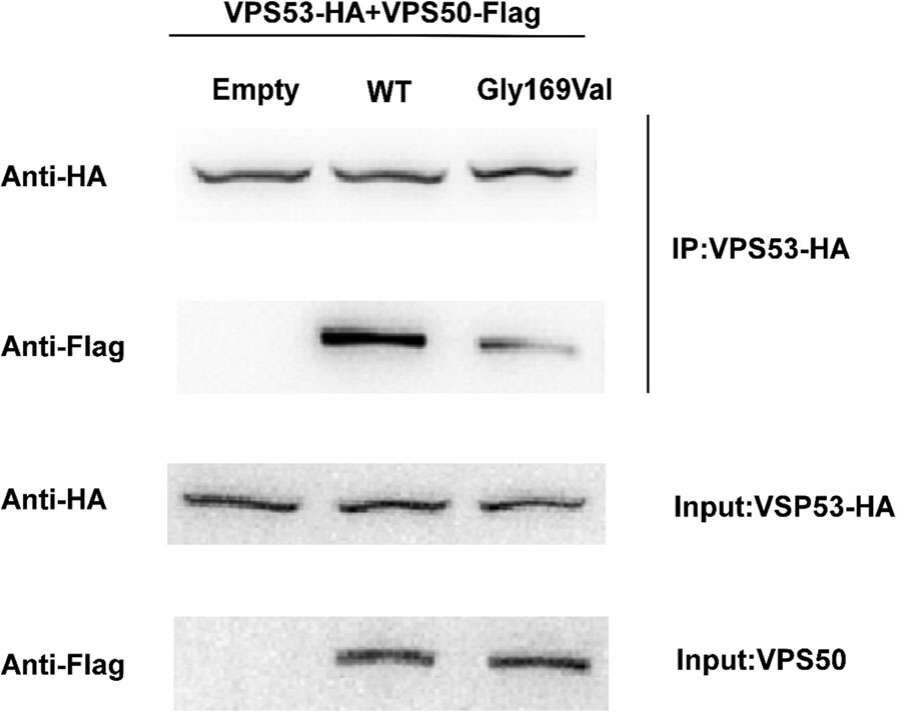
Co-Immunoprecipitation illustrated the effect of novel mutations on the physical interaction of VPS50 with VPS53. HEK293t cells were co-transfected into 4μg VPS50 plasmid constructs in pairwise combinations with 4μg VPS53 cDNA. HA immunoprecipitation, blotting with anti-Flag confirmed physical interaction between WT VPS50 and VPS53. This interaction was decreased in the p. Gly169Val mutation

Since EARP consists of ANG2, VPS52, VPS53 and VPS50, then we constructed the other three parts of EARP plasmids and tested their physical interaction by using Co-immunoprecipitation (Co-IP). We found that VPS50 could interact with VPS53 in vitro. Then, we co-transfected the various VPS50 plasmid constructs in pairwise combinations with the HA-tagged VPS53 cDNA into HEK293T cells. Immunoprecipitation with antibodies to HA and western blotting with antibodies to Flag. The results of Co-IP showed that the p. Gly169Val mutation destroyed the interaction of VPS53 to a certain extent (Fig. 3).

### Spatial expression of gene *vps50* during zebrafish early development

**Fig. 4:**
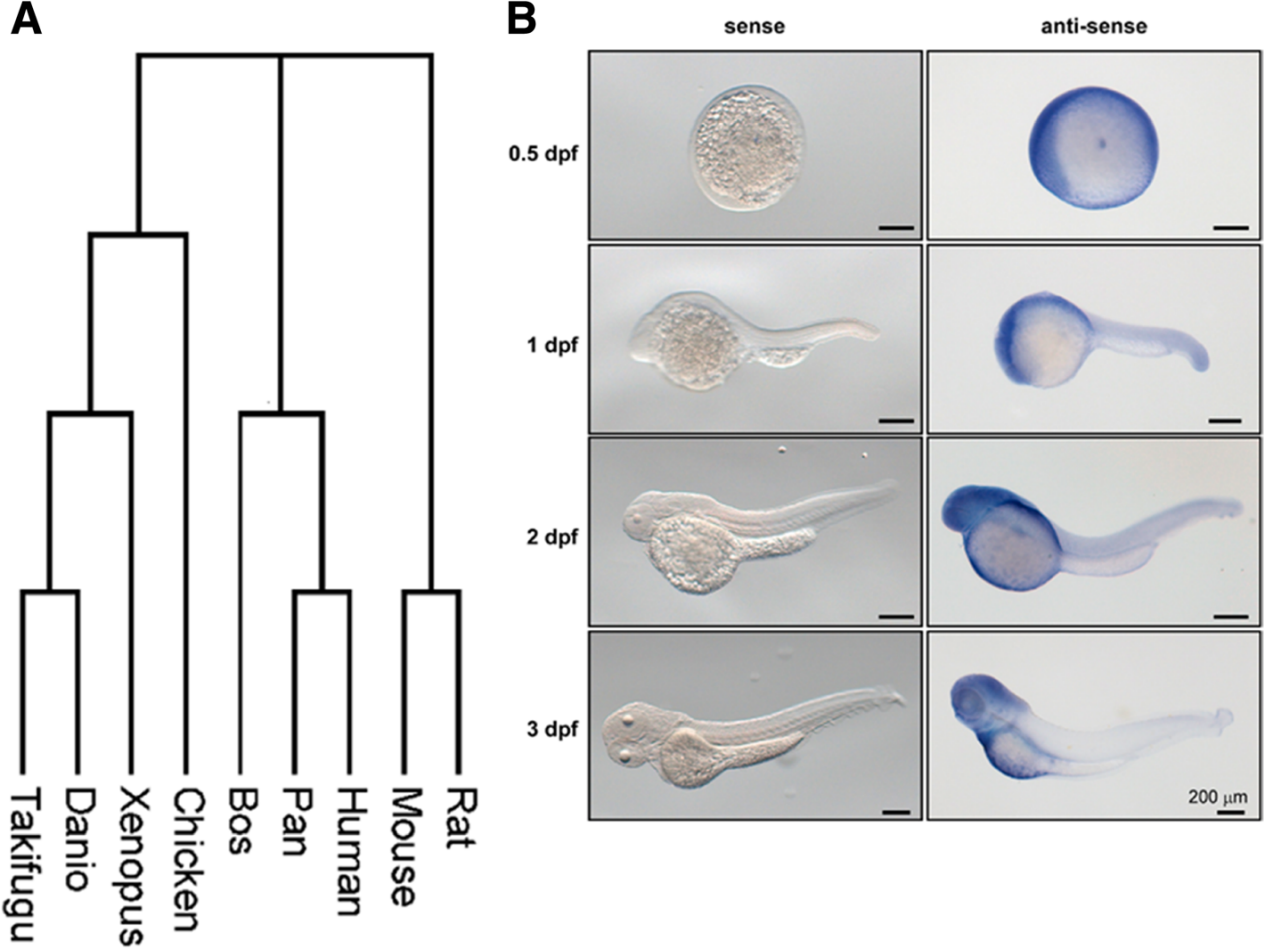
**a** A protein phylogenetic tree was constructed of obtained sequences. Zebrafish and other chordate Vps50 protein sequence were identified from the Uniprot database. Accession numbers: Human: Q96JG6; Rat: F1LSG8; Mouse: Q8CI71; Zebrafish: F1R0A4; Chicken: Q5ZKV9; Chimpanzee: H2QUX7; Bovine: F1ML08; Western clawed frog: A9UM56; Japanese pufferfish: H2UPA7. **b** Expression patterns of zebrafish *vps50* during embryogenesis. Expression of *vps50* gene was detected around the yolk sac at 1 dpf. Once the embryo reached the hatching period (2–3 dpf), *vps50* expression was seen in the head and heart (Right Figure). Representative images of *n* = 3 replicates where with each replicate having at least 15 embryos. Control images of sense strand probed on early embryos (Left Figure)

Since our research group was interested in utilizing the zebrafish model to investigate the function of conserved genes during development, we obtained 11 identified or predicted chordate Vps50 proteins spanning from the zebrafish to *Homo sapiens* to generate a phylogenic tree [15, 23]. By protein sequence alignment, a phylogenetic tree demonstrated that the Vps50 proteins seemed to follow the predicted vertebrate phylogenetic order with higher amino acid conservation being observed within a subclass or clade of animals (Fig. 4a). Interestingly, even zebrafish and humans shared common ancestors 435 million years ago, the Vps50 amino acid identity was quite conserved between zebrafish and human (~ 84.6%) (Additional file 3: Figure S2).

The high conservation of Vps50 proteins between zebrafish and human suggested that zebrafish *vps50* was a good candidate to study aspects of *VPS50* function during embryogenesis. To map the expression of zebrafish *vps50* during embryogenesis, we designed a specific sense and antisense riboprobe to determine its spatiotemporal expression by utilizing WISH. We chose the following zebrafish early stages individually: 0.5 dpf, 1 dpf, 2 dpf, and 3 dpf. The early stages tested with the antisense probe demonstrated the presence of *vps50* mRNA, while the sense probe assayed at the same stages showed no staining (Fig. 4b).

All stages tested with the antisense probe demonstrated the presence of *vps50* mRNA. By 0.5 dpf, embryonal transcripts of *vps50* were expressed globally. By 1 dpf, we started to see a tissue specific expression of *vps50*. *vps50* gene was expressed around the yolk sac, especially in the height of the head and a small amount of expression in the tail. By 2–3 dpf, the expression pattern of *vps50* became restricted in the head and heart. Our experimental results revealed the time and spatial expression pattern of *vps50* in the early development of zebrafish.

### *vps50* affecting the development of zebrafish embryos

**Fig. 5:**
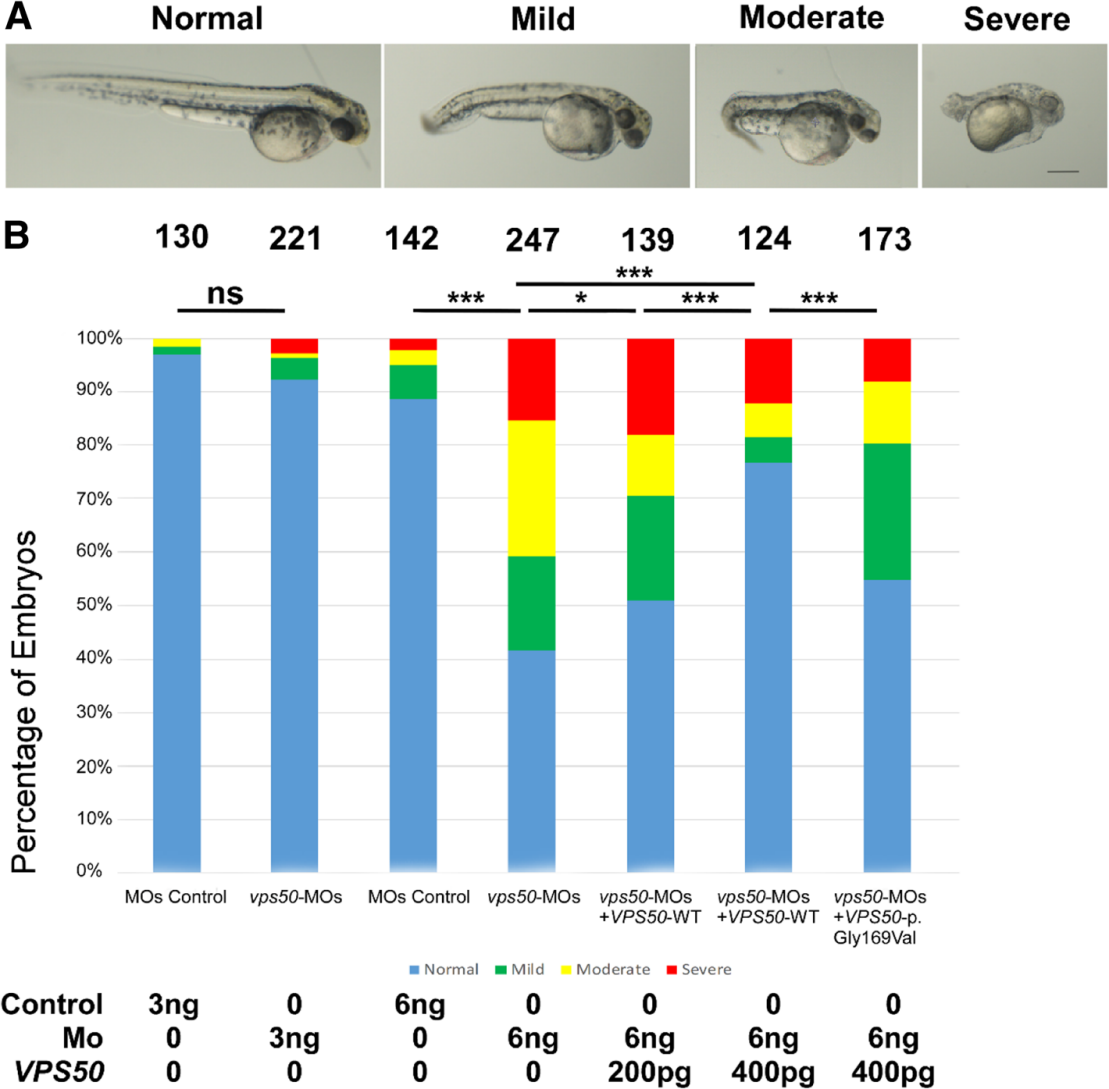
**a** Distribution of the four categories in each of the experimental groups**.** We clustered these injected embryos into four categories according to the severity of their morphology. Grade 1: WT like; Grade 2: mild, curving axis compared with WT embryos; Grade 3: moderate, up to 1/3 shortened axis; Grade 4: severe, the body axis just extended out of the range of the yolk ball. **b** Injection of *vps50*-MOs yielded high proportion abnormal phenotypes. Wild-type VPS50 exhibited higher efficiency saving ability than the p. Gly169Val mutation. Dosage-dependent rescue of vps50 knock-down zebrafish embryo with Human *VPS50* mRNA. 6 ng *vps50-*MOs were coinjected with 0, 200, and 400 pg of *VPS50* mRNA. The number above each bar was the total number of embryos counted. The *P* value was calculated by chi-squared analysis

The results of WISH showed that *vps50* was widely expressed during embryogenesis. To explore the effects of *vps50* deficiency on development of zebrafish embryos, we designed antisense MOs (*vps50*-MOs), directed against the ATG of *vps50* gene. Injection of *vps50*-MOs, and not control MOs, into embryos resulted in different populations of abnormal embryos after 48 h post fertilization. These injected zebrafish embryos were clustered into four categories according to the severity of their morphology (grade 1: WT like; grade 2: mild, curving axis compared with WT embryos; grade 3: moderate, up to 1/3 shortened axis; grade 4: severe, the body axis just extend out of the range of the yolk ball, Fig. 5a). As the concentration of the *vps50*-MOs increased, the abnormal rate increased. When injecting with 6 ng *vps50*-MOs, 58.3% of the injected fish showed abnormal phenotype (Fig. 5b). To validate *vps50*-MOs knockdown efficiencies and verify the conserved function of *VPS50*, we carried out rescue experiments. Dosage-dependent rescued of *vps50* knock-down zebrafish embryo with human *VPS50* was shown in Fig. 5b. With the dosage of 400 pg *VPS50* mRNA, most of the mild and moderate phenotypes were rescued (Fig. 5b). To further clarify the effect of the *VPS50* mutation, we coinjected 6 ng *vps50*-MOs with the *VPS50* variant mRNA. Compared to WT *VPS50*, the p. Gly169Val mutation displayed the loss-of-function effects.

## Discussion

The effect of EARP-disrupting mutations on development extends to a broad range of cellular functions. It is known that in yeast cells, the loss of other components of EARP complex causes identical phenotypes with reduced growth rates [24–26]. Recent studies also found that EARP complex could function in the maturation of dense-core vesicles and *vps50* mutations could alter neuropeptide and dense-core vesicle protein levels at synapses; Knockdown of *VPS50* expression in Hela Cells disrupted the balance of TfRs on the cytoplasmic membrane. Interestedly, *TfR*^−/−^ embryos were abnormal and died before embryonic day (E) 12.5 showing kinked neural tubes [10]. Pathogenic mutations in the subunits of EARP have been found to cause the neurological deficits in the patients due to partial loss of EARP [14]. These results raise the possibility that the rare mutations in *VPS50* may be associated with human NTDs, although *VPS50* has never been reported to be associated with any human disease.

By high-throughput sequencing, we identified a rare case-specific mutation (p. Gly169Val) in *VPS50* gene in a Chinese NTDs cohort. The p. Gly169Val mutation was located in the highly conserved residues in vertebrates, suggesting its critical function. In vitro results showed that the p. Gly169Val was a loss-of-function mutation, delaying transferrin recycling and disrupting its interaction with VPS53.

The high conservation of Vps50 proteins between zebrafish and human suggested that zebrafish *vps50* was a good candidate to study functions of *VPS50* in early embryo development. Using WISH, we firstly identified that *vps50* was highly expressed in early development of zebrafish and the expression pattern of *vps50* changed with different developmental stages. Embryonal transcripts of *vps50* were expressed globally at 0.5 dpf. After 1 dpf, the expression pattern of the *vps50* gene became more concentrated and specific. By 2 dpf and 3 dpf, the late pharyngula stage, expression of the zebrafish *vps50* gene was localized in the head and heart of the embryo. These results were consistent with the previous studies that many genes in the recycling endosome were found to be expressed throughout the early embryo [27, 28]. Since *vps50* was highly expressed in zebrafish embryonic development, *vps50* knockdown by MOs was carried out. Knockdown of *vps50* during embryo development of zebrafish resulted in high ratio of abnormal body axis. These defects could be rescued significantly by injection of exogenous human *VPS50*. Knockdown and rescue experiments suggested that the presence of *Vps50* in the early embryo might be indispensable during vertebrate development. Further rescue study in zebrafish suggested that p. Gly169Val displayed loss-of-function effects compared with wild-type *VPS50*.

## Conclusion

This is the first report describing the association of *VPS50* gene mutations with human NTDs. In vivo functional analysis demonstrated the expression pattern of *vps50* during embryogenesis and found that it played an important role in the early development of zebrafish. Further understanding the roles of *VPS50* in the regulations of post-endocytic transport pathways and development are critical and necessary.

## Additional files

Additional file 1: List of VPS50 mutations. (XLSX 68 kb)

Additional file 2: **Figure S1.** The immunofluorescence showed that after using puromycin to filter untransfected cells, transfection efficiency was quite high. (DOCX 2395 kb)

Additional file 3: **Figure S2.** Sequence alignment of different vertebrate Vps50. (DOCX 1448 kb)

## Abbreviations

EARP: Endosome-associated recycling protein
GARP: Golgi-associated retrograde protein
MOs: Morpholino oligos
NTDs: Neural Tube Defects
TfRs: Transferrin receptors
WISH: Whole mount in situ hybridization
