# Supplementary material for: The rare mutation in the endosome-associated recycling protein gene VPS50 is associated with human neural tube defects
Source: Mol Cytogenet. 2019 Feb 20;12:8. doi: 10.1186/s13039-019-0421-9 (PMC6381738; doi:10.1186/s13039-019-0421-9)
Supplement: Supplementary file 2 — Figure S1. The immunofluorescence showed that after using puromycin to filter untransfected cells, transfection efficiency was quite high. (DOCX 2395 kb) [file 13039_2019_421_MOESM2_ESM.docx]

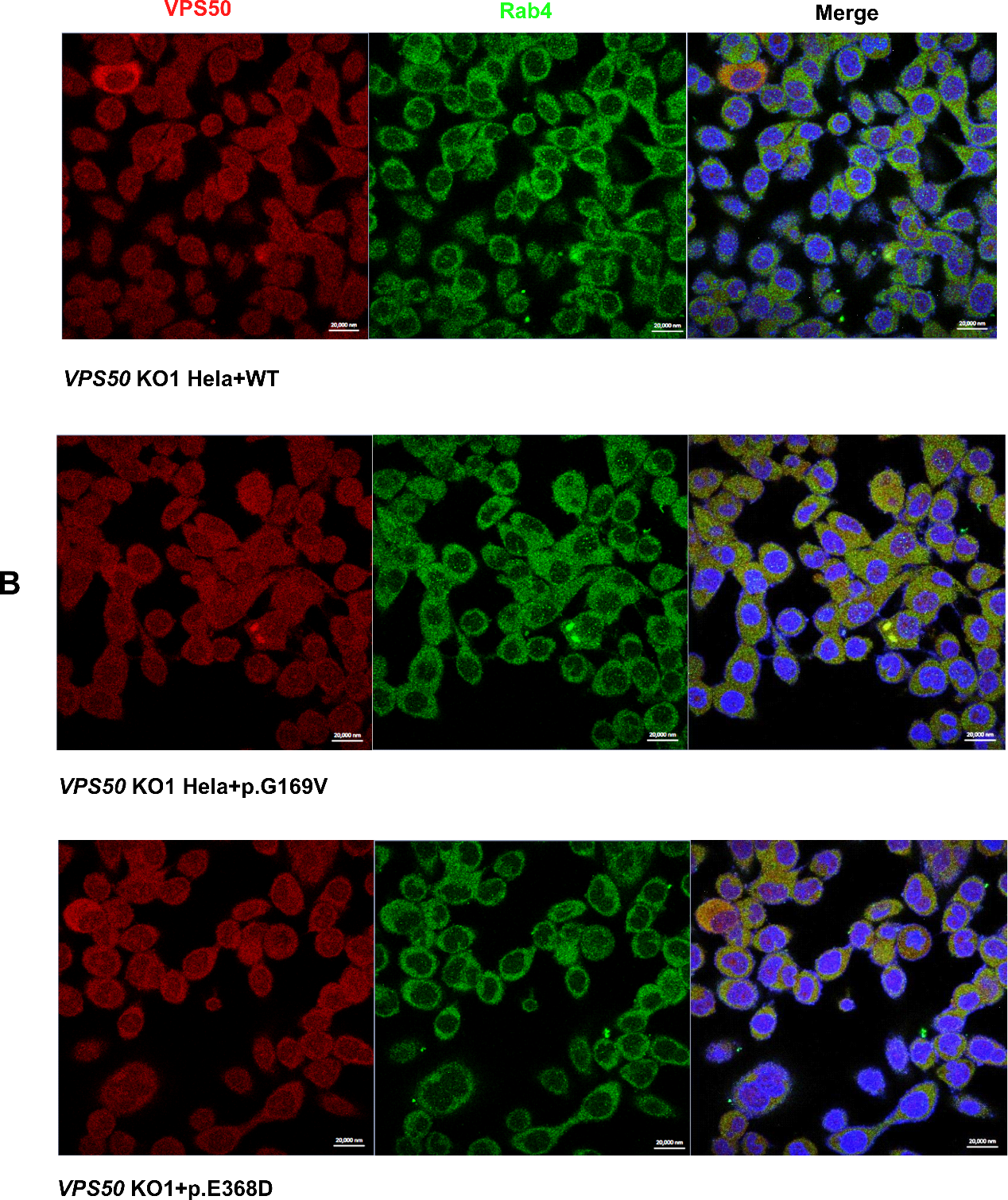


**Figure.S1.** **The immunofluorescence showed that after using puromycin to filter untransfected cells, transfection efficiency was quite high.**
