# Supplementary figures and images for: The rare mutation in the endosome-associated recycling protein gene VPS50 is associated with human neural tube defects
Source: Mol Cytogenet. 2019 Feb 20;12:8. doi: 10.1186/s13039-019-0421-9 (PMC6381738; doi:10.1186/s13039-019-0421-9)

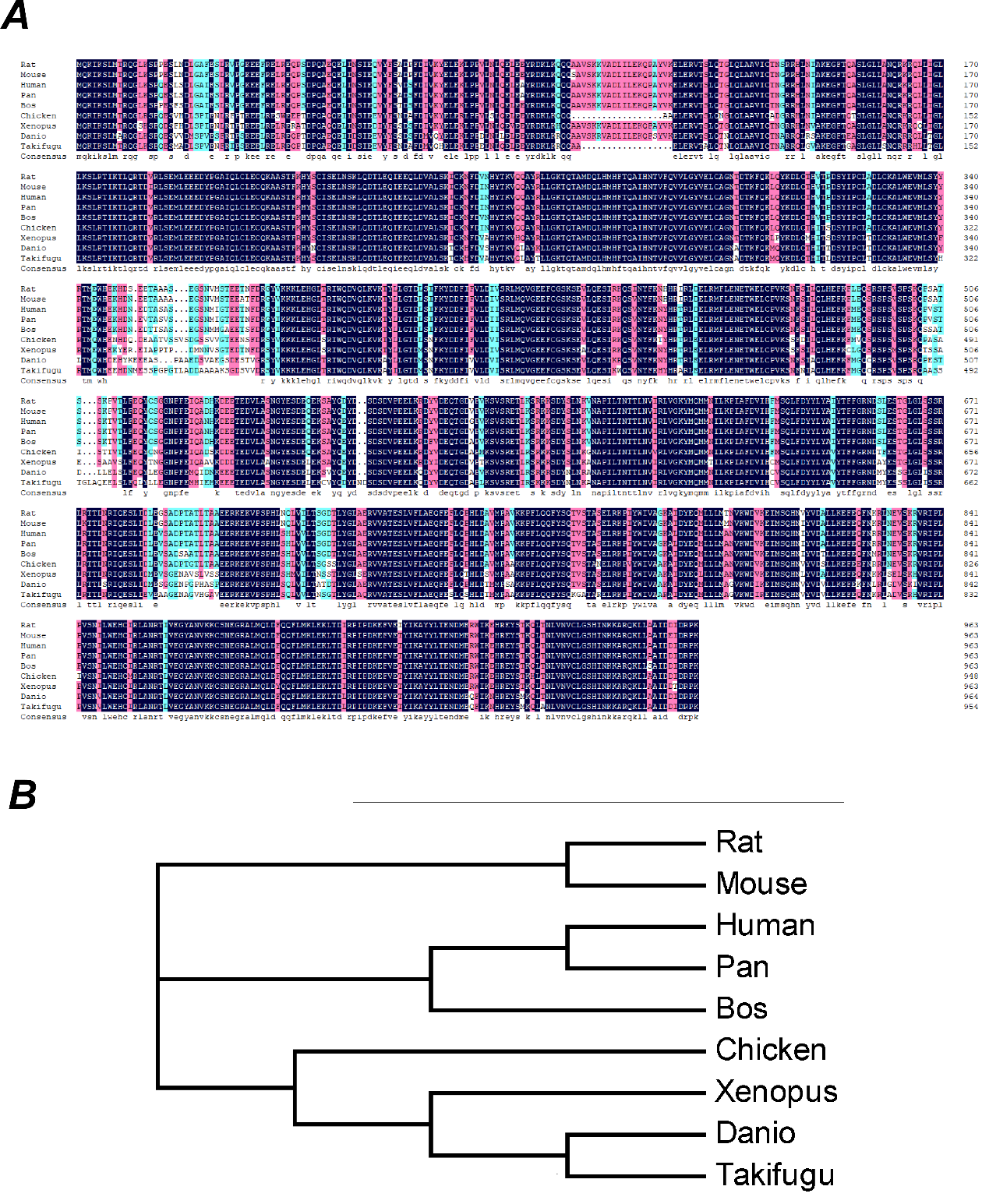


**Figure.S2. Sequence alignment of different vertebrate Vps50.**

Supplement: Supplementary file 3 — Figure S2. Sequence alignment of different vertebrate Vps50. (DOCX 1448 kb) [file 13039_2019_421_MOESM3_ESM.docx]
